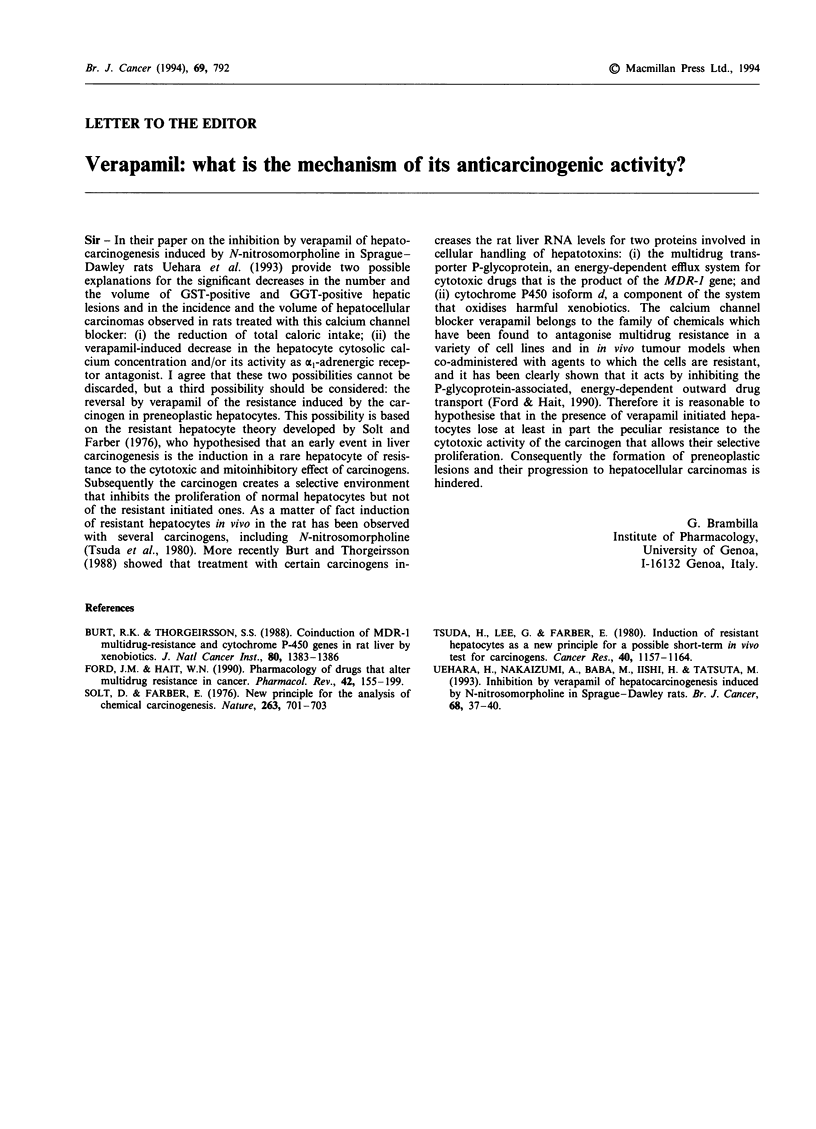# Verapamil: what is the mechanism of its anticarcinogenic activity?

**DOI:** 10.1038/bjc.1994.150

**Published:** 1994-04

**Authors:** G. Brambilla


 © Macmillan Press Ltd., 1994

**LETTER TO THE EDITOR**

### Verapamil: what is the mechanism of its anticarcinogenic activity?

**Sir** – In their paper on the inhibition by verapamil of hepatocarcinogenesis induced by *N*-nitrosomorpholine in Sprague–Dawley rats Uehara *et al.* (1993) provide two possible explanations for the significant decreases in the number and the volume of GST-positive and GGT-positive hepatic lesions and in the incidence and the volume of hepatocellular carcinomas observed in rats treated with this calcium channel blocker: (i) the reduction of total caloric intake; (ii) the verapamil-induced decrease in the hepatocyte cytosolic calcium concentration and/or its activity as $\alpha_1$-adrenergic receptor antagonist. I agree that these two possibilities cannot be discarded, but a third possibility should be considered: the reversal by verapamil of the resistance induced by the carcinogen in preneoplastic hepatocytes. This possibility is based on the resistant hepatocyte theory developed by Solt and Farber (1976), who hypothesised that an early event in liver carcinogenesis is the induction in a rare hepatocyte of resistance to the cytotoxic and mitoinhibitory effect of carcinogens. Subsequently the carcinogen creates a selective environment that inhibits the proliferation of normal hepatocytes but not of the resistant initiated ones. As a matter of fact induction of resistant hepatocytes *in vivo* in the rat has been observed with several carcinogens, including *N*-nitrosomorpholine (Tsuda *et al.*, 1980). More recently Burt and Thorgeirsson (1988) showed that treatment with certain carcinogens increases the rat liver RNA levels for two proteins involved in cellular handling of hepatotoxins: (i) the multidrug transporter P-glycoprotein, an energy-dependent efflux system for cytotoxic drugs that is the product of the *MDR-1* gene; and (ii) cytochrome P450 isoform *d*, a component of the system that oxidises harmful xenobiotics. The calcium channel blocker verapamil belongs to the family of chemicals which have been found to antagonise multidrug resistance in a variety of cell lines and in *in vivo* tumour models when co-administered with agents to which the cells are resistant, and it has been clearly shown that it acts by inhibiting the P-glycoprotein-associated, energy-dependent outward drug transport (Ford & Hait, 1990). Therefore it is reasonable to hypothesise that in the presence of verapamil initiated hepatocytes lose at least in part the peculiar resistance to the cytotoxic activity of the carcinogen that allows their selective proliferation. Consequently the formation of preneoplastic lesions and their progression to hepatocellular carcinomas is hindered.

G. Brambilla
Institute of Pharmacology,
University of Genoa,
I-16132 Genoa, Italy.

**References**

BURT, R.K. & THORGEIRSSON, S.S. (1988). Coinduction of MDR-1 multidrug-resistance and cytochrome P-450 genes in rat liver by xenobiotics. *J. Natl Cancer Inst.*, **80**, 1383–1386

FORD, J.M. & HAIT, W.N. (1990). Pharmacology of drugs that alter multidrug resistance in cancer. *Pharmacol. Rev.*, **42**, 155–199.

SOLT, D. & FARBER, E. (1976). New principle for the analysis of chemical carcinogenesis. *Nature*, **263**, 701–703

TSUDA, H., LEE, G. & FARBER, E. (1980). Induction of resistant hepatocytes as a new principle for a possible short-term *in vivo* test for carcinogens. *Cancer Res.*, **40**, 1157–1164.

UEHARA, H., NAKAIZUMI, A., BABA, M., IISHI, H. & TATSUTA, M. (1993). Inhibition by verapamil of hepatocarcinogenesis induced by N-nitrosomorpholine in Sprague–Dawley rats. *Br. J. Cancer*, **68**, 37–40.